# Barriers to the uptake of eye care services: A cross-sectional survey from rural and urban communities

**DOI:** 10.1371/journal.pone.0308294

**Published:** 2024-08-15

**Authors:** Bismark Owusu-Afriyie, Nancy Peter, Felix Ivihi, Issac Kopil, Theresa Gende

**Affiliations:** 1 Faculty of Medicine and Health Sciences, Divine Word University, Madang, Papua New Guinea; 2 The Fred Hollows Foundation NZ, Auckland, New Zealand; University of the Witwatersrand Johannesburg, SOUTH AFRICA

## Abstract

**Purpose:**

To explore the barriers to the uptake of eye care services in urban and rural communities in Papua New Guinea.

**Methods:**

This was a population-based cross-sectional descriptive study and involved multi-stage sampling. Communities were randomly selected from each of the three clusters of Madang District for free eye care outreaches from June to September 2022. A structured questionnaire was used to collect data from the outreach patients. The study excluded attendees who refused to consent. Responses were rated from 1 (not a barrier) to 10 (a very strong barrier). The *p*-value significance was set at ≤ 0.05.

**Results:**

The majority of the 972 participants (60.2%) were from rural communities. The mean age of participants was 40.82 ± 13.14 years. Almost two-thirds of the participants (61.4%) never had an eye examination before this study was conducted. All the participants reported that time constraint, insufficient income, good vision in the fellow eye, not considering their eye conditions as serious issues and cultural beliefs were personal barriers to accessing eye care services. Provider-related challenges included long waiting periods at eye clinics and fear of procedure complications. There were differences in barriers with respect to the participants’ demographic clusters.

**Conclusion:**

There are major personal- and service-related barriers to eye care services in Madang. These barriers could be overcome through strategic human resource development, health education, school screening programs, and establishing eye care centres in the communities to improve the uptake of eye care services in Madang and more widely across the country.

## Introduction

Vision loss affects over a billion people worldwide and it is associated with increased mortality and, at times, low quality of life for affected individuals [1, 2]. The uptake of timely and appropriate eye care services by people with vision problems has been a major cause of concern in several populations across the globe [3, 4]. There is usually a disconnect between available services and services utilized by patients due to barriers in different settings [5].

Studies from different settings provide insight into the economic, attitudinal, and provider-related barriers to the uptake and utilization of eye care services. Among the identified barriers are the availability of infrastructure and human resources, cost, good vision in the other eye, time constraints, transport, culture and traditional beliefs, and limited trust in the health system [1, 3, 5–10]. Such knowledge enhances service delivery in their respective settings. In 2021, Papua New Guinea (PNG) had an estimated population of 11.8 million people, with 61.6% of the population being of working age [11]. The country is divided into twenty-two geographical provinces, and each province is subdivided into districts and clusters [11, 12]. According to a national eye health survey, the prevalence of blindness in PNG is among the highest in the world [13], suggesting an urgent need for increased accessibility and utilization of eye care services in the country. However, the specific challenges faced by people in PNG when accessing eye care services have not been thoroughly investigated.

The Madang Province is the third most populated of the 22 provinces of PNG, accounting for about 6.8% of PNG’s total population [11]. Recent studies have reported a high prevalence of refractive errors [14, 15], corneal morbidities [15], and risk factors for retinal diseases [16] among ophthalmic patients in Madang Province. These conditions could be diagnosed early and treated to prevent blindness if people have access to timely and cost-effective eye care services. The province is subdivided into six districts, with Madang District being the most heavily populated [11, 12]. Over two-thirds of Madang District’s population reside in rural areas [12], but the only eye clinic in the entire province during this study, Madang Provincial Hospital Eye Clinic, is situated in the urban centre of the province.

The dependency ratio in PNG is notably high, standing at 62.4% [11]. In PNG, patients are generally required to pay for both eye care services and any necessary consumables, but some facilities like the Madang Provincial Hospital Eye Clinic, offer free care with subsidized costs for medication and spectacles. Additionally, eye care teams in the country occasionally deliver free services through community outreach programs. It is unknown whether the challenges faced by the rural population in the district are significantly different from those of the urban population when they are accessing eye care services. It is therefore important to investigate the barriers to the uptake of eye care services in Madang District and PNG. This will enable the government, policymakers, and health system managers to develop strategies to address these challenges and ultimately improve eye health across the country. This study anticipates that identifying these issues and removing or controlling them will enhance the timely presentation of oculo-visual conditions and follow-up visits for adequate and effective treatment and interventions thereby reducing the burden of avoidable blindness in PNG.

## Materials and methods

### Study setting

Madang District, accounting for 22.5% of the provincial population, is the most populous among the six districts in Madang Province, PNG. The district consists of three clusters: Ambenob Rural, Madang Urban and Transgogol Rural. Ambenob Rural cluster has the largest population with 54,038 people followed by Madang Urban cluster with 35,971 people and Transgogol Rural cluster with 20,969 people [12]. Thus, majority of the population (67.6%) live in rural areas. There are slightly more males (52.0%) than females in Madang District [12]. A detailed map of PNG, along with the population distribution of Madang province and district, is available from the National Statistical Office [12].

### Study design and sampling techniques

This was a population-based cross-sectional descriptive study, and a multi-stage sampling method was used. The cluster populations were used to calculate the minimum sample size. The sample frame was estimated as 110,978 based on the 2011 national census [12].

The expected minimum sample size was calculated based on the Cochran formula: n_o_ = (Z^2^pq) / e^2^ where; n_o_ is the required sample size; Z is the statistic corresponding to a level of confidence set to 1.96; p is the expected proportion of the population which does not seek timely eye care. In this case, we used 56.2% per recent study on the epidemiology of eye diseases among patients reporting at the eye clinic in the district [15]; e is the desired level of precision set to 5%; q is 1 –p = 0.4381. The minimum sample size (~ 378 participants) was distributed among the clusters.

Four communities were randomly selected from each cluster for free eye care outreaches from June 2022 to September 2022. The communities were notified by community health workers and community leaders at least a week before the outreach and attendance was voluntary. The outreach centre in each community was a place readily accessible to all people in that community. Due to an initial low attendance in Ambenob Rural cluster, additional outreaches were held in four other centres in that cluster.

### Inclusion and exclusion criteria

All attendees of the community outreaches were invited to participate in the study and only those who consented were asked to complete the questionnaire. The study excluded 18 patients who refused to give consent.

### Ethical consideration

The Faculty of Medicine and Health Sciences Research Committee (FMHSRC) of Divine Word University approved this study and allocated the FMHSRC approval number FRC/MHS/59-22. Written informed consent was obtained from each participant before completing the questionnaire. Assent was obtained from minors (≤17 years old), and their parents or guardians were asked for informed consent and to help complete the questionnaire with their child(ren). The investigators followed the principles of the Declaration of Helsinki.

### Data collection procedure

A structured questionnaire was used to collect the data. The questionnaire was designed based on other similar studies [1, 3, 5–10]. It was available in two languages: English and Tok Pidgin. The questionnaire consisted of three parts: the first part assessed the participants’ demographic background; the second portion determined patient-related barriers and the final aspect assessed service-related barriers. The questionnaire was self-administered; however, for participants who could not read, investigators Nancy Peter, Felix Ivihi, and Issac Kopil read the questions and recorded the participants’ responses. Responses were rated from 1 (not a barrier) to 10 (a very strong barrier).

### Data management and analysis

Data were analyzed using IBM Statistical Package for Social Sciences for Windows, version 27.0 (IBM Corporation). *P* ≤ 0.05 was considered statistically significant. Ordinal data was presented as median and interquartile ranges, and continuous data as mean ± standard deviation. Associations were determined using Kruskal-Wallis test followed by Bonferroni correction for multiple comparisons.

## Results

### Socio-demographic features of participants

Nine hundred and seventy-two out of the 990 outreach attendees consented to participate in the study. The response rate was 98.2%. There were more female participants (50.9%) than males. Majority of the participants (60.2%) were rural dwellers, and the mean age of participants was 40.82 ± 13.14 years (ranging from 13 to 80 years). Most of the participants had only primary education (40.9%), and 118 (12.1%) had no formal education. Nearly two-thirds of the participants (61.4%; 298 males and 299 females) had never checked their eyes prior to the outreach, and only 217 (22.3%) had an eye check within two years prior to this study. The most commonly reported occupations were trading (33.2%), farming (18.0%), and housewife duties (12.7%). The distribution of the participants’ demographics is shown in Table 1.

**Table 1:** Demographics of participants.

Characteristics	Participants; n (%)	Characteristics	Participants; n (%)
**Gender**		**Last Eye Examination**	
Male	477 (49.1)	≤ 1 year ago	138 (14.2)
Female	495 (50.9)	1–2 years ago	79 (8.1)
**Residence/Cluster**		2–5 years ago	92 (9.5)
Ambenob Rural	231 (23.8)	6–10 years ago	28 (2.9)
Transgogol Rural	354 (36.4)	10–20 years ago	19 (2.0)
Madang Urban	387 (39.8)	>20 years ago	19 (2.0)
**Age Group (years)**		Never	597 (61.4)
11–20	43 (4.4)	**Primary Occupation**	
21–30	224 (23.0)	Retail trader	323 (33.2)
31–40	254 (26.1)	Farmer	175 (18.0)
41–50	233 (24.0)	Housewife	123 (12.7)
51–60	148 (15.2)	Student	84 (8.6)
61–70	63 (6.5)	Customer care representative	35 (3.6)
Above 70 years	7 (0.7)	Security person	31 (3.2)
**Level of Education**		Health worker	30 (3.1)
Primary	398 (40.9)	Teacher/lecturer	29 (3.0)
Secondary	282 (29.0)	Artisans	27 (2.8)
Tertiary	174 (17.9)	Accountant	24 (2.5)
No formal education	118 (12.1)	Retired	21 (2.2)
		Others*	70 (7.2)

*human resource officer-1; clergy-16; secretary-6; engineer-14; magistrate-2; driver-10; politician-3; director/manager-11; unemployed-5; not reported-2.

### Patient-related barriers

**Fig 1:**
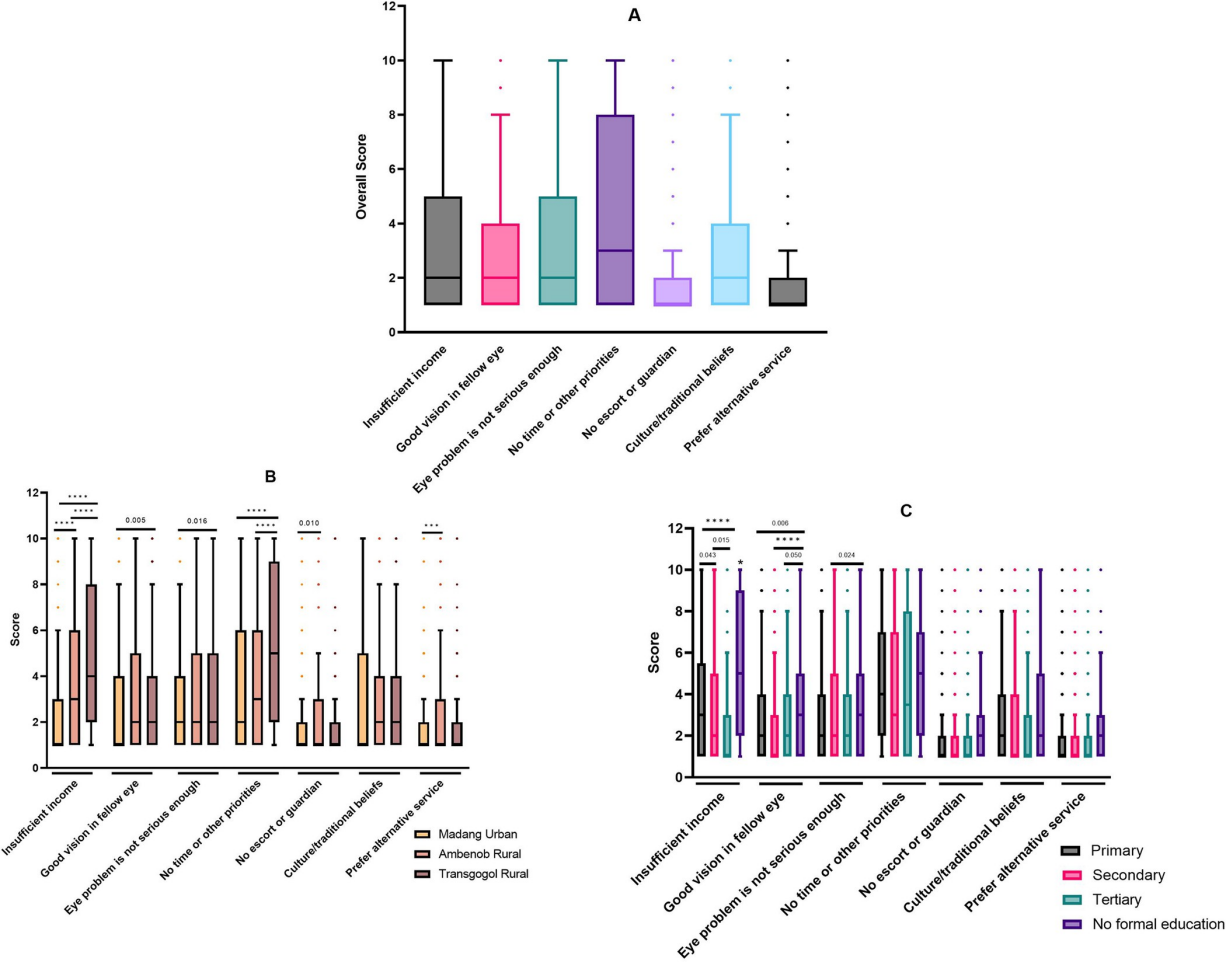
Participants’ personal barriers to the uptake of eye care services in Madang District. Responses were rated from 1 (not a barrier) to 10 (a very strong barrier). Centre lines indicate the medians; box limits indicate the 25th and 75th percentiles; whiskers extend 1.5 times the interquartile range. The overall personal barriers to the utilization of eye care services were determined (A) and further analyzed based on the participants’ cluster (B) and educational level (C). Comparisons were determined using Kruskal-Wallis test (B and C) with Bonferroni correction for multiple tests. *p < 0.005 compared to all other groups. ***p < 0.005. ****p < 0.001. Dots indicate outliers.

The study sought to identify the personal barriers that hindered participants from accessing eye care services. Overall, the highest recorded personal barrier was their busy schedule (median = 3/10). The median responses to insufficient income, good vision in the fellow eye, eye problems not considered as a serious issue, and culture/traditional beliefs were similar (median = 2/10). The details of the overall patient-related barriers are displayed in Fig 1A.

The next step was to determine if there were any differences in responses among the three clusters (Fig 1B). The main reported barriers in Madang Urban cluster were no time or other priorities and eye problems not being serious enough to require an eye check (each median = 2/10). In Ambenob Rural cluster, insufficient income or cost and inadequate time were the most important personal barriers (each median = 3/10). Similarly, participants from Transgogol Rural cluster reported time constraints (median = 5/10) and cost (median = 4/10) as the main personal challenges to seeking eye care services. The median concern for cost and time constraints were significantly higher in the rural clusters compared to Madang Urban cluster (all *P* < 0.001). In addition, participants from Ambenob Rural cluster significantly preferred to use alternative services than those in Madang Urban cluster (*P* = 0.007). There was no statistically significant difference in all responses between males and females (all *P* > 0.05).

In terms of the influence of education on personal barriers (Fig 1C), the participants without any formal education indicated that insufficient income or cost and other priorities were the most important barriers to them (all median = 5/10). Similar responses were obtained from participants who completed only primary education (median = 3/10, and 4/10, respectively). Secondary- and tertiary-educated participants reported time constraints (median = 3/10 and 3.5/10, respectively) as their main barrier to seeking eye care services. Participants without formal education showed significant concern about cost (all *P* < 0.005), and not checking their eyes because the fellow eye had good vision compared to responses from primary, secondary, and tertiary educated participants (Fig 1C; *P* = 0.006, *P* = 0.050 and *P* < 0.001 respectively).

### Service-related barriers

**Fig 2:**
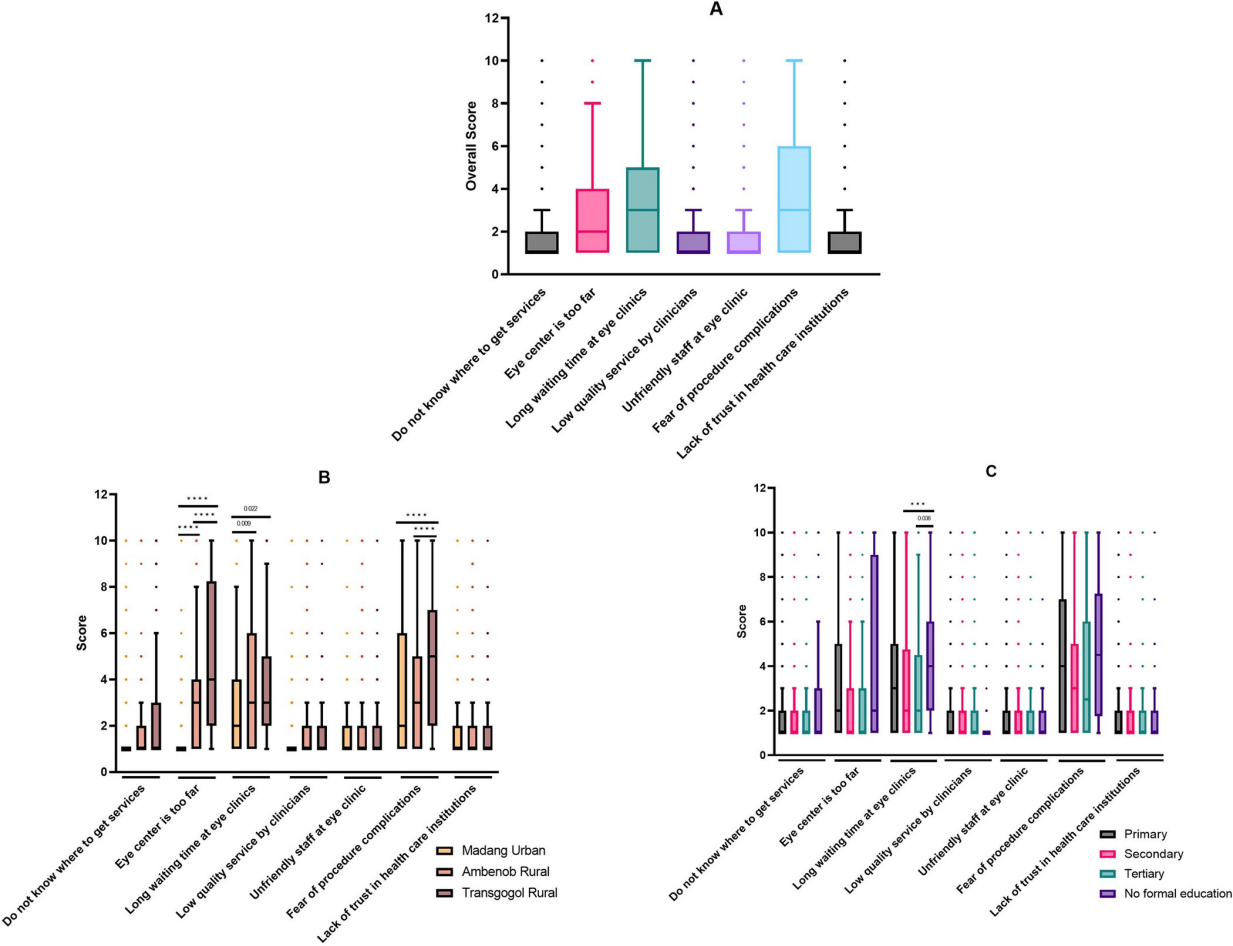
Service-related barriers to the uptake of eye care services in Madang District. Responses were rated from 1 (not a barrier) to 10 (a very strong barrier). Centre lines indicate the medians; box limits indicate the 25th and 75th percentiles; whiskers extend 1.5 times the interquartile range. The overall provider-related barriers to the utilization of eye care services were determined (A) and further analyzed based on the participants’ cluster (B) and educational level (C). Comparisons were determined using Kruskal-Wallis test (B and C) with Bonferroni correction for multiple tests. ***p < 0.005. ****p < 0.001. Dots indicate outliers.

Among all the participants, the main issues at the service level were long waiting periods at eye clinics (median = 3/10), fear of procedure complications (median = 3/10) and the distant location of eye centres (median = 2/10). See Fig 2A for details.

From Fig 2B, the participants from Transgogol Rural cluster indicated that the fear of procedure complications (median = 5/10) and the long distance to eye clinics (median = 4/10) were their major barriers at the provider level. Ambenop Rural participants reported the distant location of eye clinics, long waiting time at eye clinics, and fear of procedure complications as the main service-related barriers (each median = 2/10). Participants from Madang Urban cluster also reported long waiting time at eye clinics and fear of procedure complications as the main factors affecting access to eye care services (each median = 2/10). Participants from the rural clusters (Ambenob and Transgogol) were significantly concerned about the distant location of eye clinics (all *P*<0.001) and the long waiting time at eye clinics compared to participants from the urban cluster (*P* = 0.009 and 0.022 respectively). Participants from Transgogol Rural cluster were also more concerned about procedure complications compared to the other two clusters (all *P*< 0.001). There was no significant difference in the service-related barriers reported by male and female participants.

From Fig 2C, the participants without any formal education indicated that the fear of procedure complications (median = 4.5/10) was the most important barrier to them followed by long waiting time at eye clinics (median = 4/10). Similar responses were obtained from participants who completed only primary education (median = 4/10 and 3/10, respectively). Among the secondary- and tertiary-educated participants, fear of procedure complications (median = 3/10 and 2.5/10, respectively) was the main obstacle. Participants without formal education significantly considered long waiting periods at eye clinics as a challenge compared to those with secondary (*P* = 0.001) and tertiary education (*P* = 0.038).

### Further perspectives on barriers to accessing eye care services

The study participants were asked to provide any additional comments about the barriers to the uptake of eye care services. The responses were similar to the above findings on patient-related and service-related barriers. Four participants (0.4%) indicated that the fear of eye surgery was their barrier while one participant (0.1%) indicated that the use of lemon grass to treat red eyes was the reason for not visiting an eye clinic for treatment. In addition, 55 participants (5.7%) indicated that they were grateful for the outreach services and 10 participants (1.0%) suggested the need for advocacy and health promotion. Details of the participants’ comments are shown in Table 2.

**Table 2:** Further perspectives on barriers to accessing eye care services.

Comment	Participant; n (%)
**Patient-related barriers**	
Traditional beliefs	32 (3.3)
Good vision in one eye is a barrier	13 (1.3)
Busy schedule	12 (1.2)
Not interested in eye checks	3 (0.3)
Alternative treatment	2 (0.2)
No comments	910 (93.6)
**Service-related barriers**	
Fear of surgery, light, and other procedure complications	33 (3.4)
Need for advocacy and health promotion	10 (1.0)
Poor accessibility to eye clinics	8 (0.8)
Need better health workers and clinics	8 (0.8)
Unfriendly staff at eye clinics	1 (0.1)
No comments	857 (88.2)

## Discussion

In this study, the participants’ demographics had significant influence on the kind of barriers they encounter when they are assessing eye care services in the district. Majority of the participants (60.2%) were from rural communities. This is similar to the population distribution in the district. More than half (67.6%) of residents in the Madang District live in rural communities [12]. However, there were more female participants (50.9%) in this study compared to the relatively greater proportion of males (52.0%) in the district. Studies from India and China have reported that females seek less health care than males even if the females have poorer vision [17–19]. However, females attend more free healthcare services than males probably due to the associated cost [20, 21]. This gender imbalance in health care uptake needs to be addressed with the necessary enablers in different populations. The minimum age of the participants in this study was 13 years. This suggests that the utilization of eye care services by children in the district was poor. A study in Madang Province reported a low uptake of refractive error services among children [14]. School vision programs may be vital to detect and treat eye problems among children in the district [15].

Strikingly, most of the participants (61.4%) had never checked their eyes prior to the outreach. Even though this rate is lower than that of the TREE study [22], it is alarming since there is high prevalence of potentially blinding conditions such as diabetic retinopathy, age-related macular degeneration, cataract, uncorrected refractive errors and corneal problems in Madang, and across PNG [13–15, 23]. A recent study by Owusu-Afriyie et al also indicated that there was high prevalence of diabetes, and hypertension among ophthalmic patients in Madang Province, and these are risk factors for retinal diseases [16]. Therefore, regular and periodic eye examinations are crucial for early detection and intervention to preserve sight and prevent blindness in the district. Maintaining good eye health may go a long way to make the participants more productive, boost their socio-economic status, strengthen the PNG economy and help in achieving the United Nations’ sustainable development goals [24], since almost all the study participants (97.1%) were in active employment. It is necessary to increase the availability of eye care services to the public, and community members should be made more aware of the importance of good eye health to increase their timely access to available services.

In general, the participants reported low levels of personal barriers to the uptake of eye care services. Chief among their personal struggles was their busy schedules. This may be attributed to their engagement in active occupations and the fact that the only eye clinic in the district is open during the traditional working hours (that is, from 8:00 am to 4:00 pm) and does not open on evenings, weekends, and holidays. People employed in the regular working hours often find it difficult to get free time to attend to their health needs [25]. One way to overcome this barrier is for employees to negotiate with their supervisors for free periods or breaks to seek health care services. In this way, employers can also benefit from increased productivity and growth when their workers are in good health. Eye care service providers are strongly encouraged to offer weekend or extended-hours services and initiate workplace outreach programs to increase the uptake of eye care services among individuals who are primarily occupied by employers during traditional working hours.

Participants from Madang Urban cluster reported that they did not consider their eye problems as serious conditions to warrant attention. A comparable finding was reported in the Andhra Pradesh eye disease study in India. In that study, Marmamula and colleagues noted that about a third of their study participants did not perceive their eye problems as serious issues, hence they did not seek eye care services [26]. This is concerning as many ocular and visual conditions remain asymptomatic until the diseases have advanced [27]. Additionally, an eye disease could be the manifestation of a life-threatening systemic disease such as diabetes, hypertension, systemic lupus erythematosus and AIDS [28]. To address this, the public, especially residents of Madang Urban cluster, need greater awareness that eye examinations are valuable not just for their eyesight but also for their general health. It is crucial for individuals to undergo regular eye examinations and seek professional care at the first sign of any eye-related issues.

On the other hand, participants from Ambenob Rural and Transgogol Rural clusters, likewise participants without any formal education were more concerned about financial issues. These identified geographic and demographic differences in the personal barriers are similar to reports from other studies [25, 29–32]. In Andhra Pradesh state of India, financial constraint was the most frequently cited reason for not seeking eye care services [26]. In Oyo and Obun states of Nigeria, patients and health workers indicated that cost and poor knowledge about glaucoma were the main problems leading to the poor uptake of glaucoma services [31]. In Scotland, there was a rise in the utilization of eye care services especially among those with higher education and high-income earners after the implementation of a free eye care policy [30]. Islam et al have suggested that low-cost small incision cataract surgeries could make cataract surgery more accessible to patients in Dhaka, Bangladesh [33]. More recently in 2022, Atta et al reported that medical cost and insurance issues were the main barriers to eye care services among their participants in Pittsburgh [25].

The cost of eye care includes the service fees, consumables, medications, transport, food, and accommodation for patients and guardians who may travel from long distances. The eye clinic in Madang District is situated in Madang Urban cluster, therefore residents of Ambenob Rural and Transgogol Rural clusters have to travel longer distances and spend more time and money to access eye care services compared to those living in the urban area. Even though non-governmental and charity organizations offer free services and subsidize consumables at the eye clinic, the other associated costs may be unbearable for people with low incomes in rural communities. Hence, the creation of income-generation enterprises in the rural communities of Madang District may enhance the socio-economic well-being of the residents and improve their healthcare-seeking habits.

Again, culture and/or traditional beliefs and having good vision in the other eye were barriers to the uptake of eye care services by participants from Ambenob Rural and Transgogol Rural clusters. In the rapid assessment of visual impairment project in South India, 2.6% of the participants reported good vision in the unaffected eye as a barrier to the uptake of eye care services [3]. While good vision in one eye could be adequate to perform daily activities, monocular vision can negatively impact a person’s quality of life [34]. Individuals with good vision in only one eye may fail to accurately judge distances and have restricted field of view on the affected side [35]. In addition, the principal causes of visual impairment and blindness such as cataracts, refractive errors, corneal scarring and opacities, diabetic retinopathy, age-related macular degeneration, and other posterior segment diseases [13, 15] are usually bilateral conditions that progress faster in one eye than the other. Thus, having good vision in one eye does not necessarily suggest the absence of morbidity in that eye. Community health education is important to address these barriers, especially in Ambenob Rural and Transgogol Rural clusters as well as other similar settings across the globe.

At the service level, all the study participants were concerned about the long waiting periods at eye clinics, fear of procedure complications and the distant location of eye centres. At the time of this study, there were two ophthalmologists, no optometrist and six ophthalmic clinicians serving the entire district. Madang District has a widely dispersed population and transport is a challenge for many communities. Thus, the number of eye care professionals in the district is inadequate for effective coverage of eye care services. Stakeholders should therefore plan and increase the training of more eye care professionals and specialists for the district. Adding primary eye care services to the already established primary health care system may also decrease this burden and increase accessibility. In the meantime, local nurses, community health workers and teachers could be trained to identify sight-threatening oculo-visual conditions and refer them to the available facilities for further assessment and management. These collaborative measures could reduce the waiting time at eye clinics, late reporting of oculo-visual conditions and cost of seeking eye care services [36–38]. While trust is a key barrier to eye care particularly among underutilized groups [39], the concerns about procedural complications in the current study are not likely due to trust or low quality of services (see Fig 2). It is anticipated that increased efforts towards demystifying eye care procedures, along with highlighting the benefits of eye examinations and treatments, including surgeries, will boost public confidence and lead to a higher uptake of eye care services in the district.

The distant location of the only eye clinic in the district is a significant hindrance to the uptake of eye care services by residents in the two rural clusters. As evidenced in this study, additional outreaches were organized in Ambenob Rural cluster to increase the accessibility and patronage of the free eye care services. Even though this is the largest cluster by population and additional outreach centres were added compared to the other clusters, it recorded the lowest number of participants. As discussed previously, Madang District has a widely dispersed population and transport is a challenge in many rural communities. It is firmly recommended that healthcare authorities in the district intensify awareness programs about eye care in remote communities. In addition, stakeholders of eye care in the district should plan and make eye care services more readily accessible in rural communities through the establishment of health posts, outreach camps, and mobile clinics.

Contrary to findings from suburban communities in Mozambique [40], there was a low reported use of alternative treatments and traditional beliefs in the current study. Nonetheless, such practices–like using lemon grass to treat red eyes–should be properly addressed through targeted health education and advocacy programs in the district. This approach can also increase the uptake of services such as cataract surgery [41].

In this study, 61.4% of the participants had the opportunity to receive their first eye examination when free services and consumables were offered, along with bringing eye care services directly to their communities. These are people who might not have otherwise sought eye care. Similarly, Akuffo et al reported that 73.4% of their study participants in South Africa had never had an eye examination before the study [42]. Sengo et al also indicated that 41.7% of participants with eye symptoms in Nampula (Mozambique) had not sought eye care before their study [40]. All these findings point to an urgent need to improve accessibility and utilization of eye care services, especially in low- and middle-income countries.

### Summary and opportunities for future research

In summary, the study identified patient- and service-related barriers to the utilization of eye care services in Madang District. Paramount among the barriers were time constraints, long waiting periods at eye clinics, the distant location of eye clinics, and fear of procedure complications. Rural participants were further concerned about cost, traditional beliefs, and having good vision in the other eye. Future studies and public health education programs should explore and address the specific traditional beliefs/practices in the communities and the fear of eye care procedures to increase the uptake of services in the district and country. The findings of this study have the potential to guide the government, other stakeholders, and providers of eye care services in PNG and other countries. Removing these barriers through strategic human resource mobilization, health education, and the creation of more eye care centres that offer affordable services with extended working hours would improve the uptake of eye care services in Madang and across PNG.

In this study, the questionnaire was given solely to the outreach attendees in the communities, which could result in a selection bias. Nonetheless, this may have little effect due to the random sampling of the communities and the large number of participants in the study. The high attendance at the outreach services and active participation in this study may be attributed to the accessibility and proximity of the outreach centres to the people, the free services and consumables offered, and a need for eye care services in the communities.

## Supporting information

S1 File Questionnaire for the study. (DOCX)

S2 File Raw data for the study. (XLSX)
