# Supplementary material for: Barriers to the uptake of eye care services: A cross-sectional survey from rural and urban communities
Source: PLoS One. 2024 Aug 15;19(8):e0308294. doi: 10.1371/journal.pone.0308294 (PMC11326634; doi:10.1371/journal.pone.0308294)
Supplement: S1 File — (DOCX) [file pone.0308294.s001.docx]

**Questionnaire**

**Part A: Socio-demographic Information**

Gender: M / F Age:­­­______ Occupation:_______________ Location: ­­­­­­­­­­­­­­­­­­­­­­ Urban Rural

Please select the cluster in Madang District that you belong to.

Ambenob Rural Madang Urban Transgogol Rural

Level of Education: None Primary Secondary Tertiary

When was the last time you visited an eye clinic? ____________________________________

**Part B: Person-related Barriers**

1. Please indicate (by ticking the appropriate box) if any of these is a barrier to your uptake of eye care services and to what extent (where **1 means that it is not a barrier** at all and **10 means that it is a very strong barrier**).

|  | 1 | 2 | 3 | 4 | 5 | 6 | 7 | 8 | 9 | 10 |
| --- | --- | --- | --- | --- | --- | --- | --- | --- | --- | --- |
| Insufficient income (cost) |  |  |  |  |  |  |  |  |  |  |
| Good vision in the fellow eye |  |  |  |  |  |  |  |  |  |  |
| Eye problem is not serious enough |  |  |  |  |  |  |  |  |  |  |
| No time / other priorities / too busy |  |  |  |  |  |  |  |  |  |  |
| No escort or guardian to help |  |  |  |  |  |  |  |  |  |  |
| Culture/traditional belief |  |  |  |  |  |  |  |  |  |  |
| Prefer to use alternative service |  |  |  |  |  |  |  |  |  |  |

| Any additional comments (including alternative services): |
| --- |

**Part C: Service-related Barriers**

1. Please indicate (by ticking the appropriate box) if any of these is a barrier to your uptake of eye care services and to what extent (where **1 means that it is not a barrier** at all and **10 means that it is a very strong barrier**).

|  | 1 | 2 | 3 | 4 | 5 | 6 | 7 | 8 | 9 | 10 |
| --- | --- | --- | --- | --- | --- | --- | --- | --- | --- | --- |
| Do not know where to get services |  |  |  |  |  |  |  |  |  |  |
| Eye centers/clinics are too far |  |  |  |  |  |  |  |  |  |  |
| Long waiting time at eye clinics |  |  |  |  |  |  |  |  |  |  |
| Low quality of care by eye doctors and clinicians |  |  |  |  |  |  |  |  |  |  |
| Eye clinic staff are unfriendly |  |  |  |  |  |  |  |  |  |  |
| Fear of procedure complications |  |  |  |  |  |  |  |  |  |  |
| Lack of trust in health care institutions |  |  |  |  |  |  |  |  |  |  |

| Any additional comments: |
| --- |

*********************End of questionnaire. Thank you************************
